# Brominated and Chlorinated Flame Retardants: The San Antonio Statement

**DOI:** 10.1289/ehp.1003088

**Published:** 2010-12

**Authors:** Linda S. Birnbaum, Åke Bergman

**Affiliations:** Director, NIEHS and NTP, National Institutes of Health, Department of Health and Human Services, Research Triangle Park, North Carolina, E-mail: birnbaumls@niehs.nih.gov; Professor in Environmental Chemistry, Board Member of the International Panel on Chemical Pollution, Department of Materials and Environmental Chemistry, Stockholm University, Stockholm, Sweden, E-mail: ake.bergman@mmk.su.se

The “San Antonio Statement on Brominated and Chlorinated Flame Retardants” addresses the growing concern in the scientific community about the persistent, bioaccumulative, and toxic properties of brominated and chlorinated organic flame retardants (BFRs and CFRs, respectively) and the exposure to humans and wildlife as a result of intensive use. Nearly 150 scientists from 22 countries have signed the statement since it was presented at the 30th International Symposium on Halogenated Persistent Organic Pollutants (Dioxin 2010), held 12–17 September 2010 in San Antonio, Texas. The scientist signatories are experts on the health effects and environmental fate of BFRs and CFRs and environmental contaminants in general. The International Panel on Chemical Pollution (IPCP), an international network of scientists working on various aspects of chemical pollution, also has approved the statement.

The San Antonio Statement addresses the behavior of chemicals that first appeared in the scientific literature in the 1970s. In 1973, an accidental, severe, and tragic mix-up in Michigan substituted the commercial BFR Firemaster BP-6 for magnesium oxide in cattle feed (Fries 1985). The active chemicals in Firemaster BP-6 were polybrominated biphenyls (PBBs), flame retardant chemicals similar to polychlorinated biphenyls (PCBs), but containing bromine instead of chlorine. The accidental use of PBBs led to environmental contamination affecting wildlife and humans. Although banned for several decades, PBBs can still be detected in environmental samples worldwide. Another flame retardant, tris(2,3-dibromopropyl) phosphate, commonly known as “Tris” and widely used in children’s sleepwear in the 1970s, raised concern when it was identified as a mutagen and carcinogen and was subsequently prohibited from use in sleepwear (Blum et al. 1978).

After PBBs were restricted, the use of polybrominated diphenyl ethers (PBDEs) as flame retardants in consumer products increased dramatically over the next several decades. PBDEs are structurally similar to both PCBs and PBBs and have the potential for similar behavior. However, in 2004 two commercial mixtures—PentaBDE and OctaBDE (the name reflecting the average number of bromines present)—were banned in the European Union (Cox and Efthymiou 2003) and voluntarily withdrawn from production by the sole U.S. manufacturer (Great Lakes Chemical 2009). PBDEs contained in these two mixtures were subsequently adopted as persistent organic pollutants (POPs) by the Stockholm Convention (Stockholm Convention Secretariat 2010). The cause for concern is now well recognized. However, the resistance to degradation continues to result in high concentrations of PBDEs in the environment, wildlife, and people (de Wit et al. 2006; Frederiksen et al. 2009; Su et al. 2007). The most heavily brominated mixture, DecaBDE, which is dominated by the fully brominated diphenyl ether, is currently produced and widely used in products. DecaBDE has been restricted in the European Union (European Parliament and the Council of the European Union 2003) and will be voluntarily withdrawn in the United States in 2013 (U.S. Environmental Protection Agency 2010), but production and use continue in other regions.

New BFRs and CFRs have emerged as substitutes for PBDEs or for use in other types of products. Many of these substances also are persistent and bioaccumulative and are found not only in environmental samples and house dust (Harrad et al. 2010) but also in people (Frederiksen et al. 2009) and wild-life, even those located far from the original source (de Wit et al. 2006; Montie et al. 2010). Adequate toxicity information is lacking, but data indicate that the group contains compounds that are carcinogens, mutagens, reproductive and developmental toxicants, neurotoxicants, and endocrine disruptors (Birnbaum and Staskal 2004; Darnerud 2008). Despite these properties, only a few have been regulated. There is growing evidence that specific compounds mentioned in the San Antonio Statement, such as chlorinated Tris, hexabromocyclododecane, decabromodiphenyl ethane, bis(2,4,6-tribromophenoxy) ethane, bis(2-ethylhexyl) tetrabromophthalate, Dechlorane Plus, polychlorinated alkanes, and others may be of environmental and health concern.

Currently used BFRs include hexabromocyclododecane and decabromodiphenyl ethane, both of which are used in high volumes and possess many properties (both environmental and biological) similar to those of PBDEs. Chlorinated Tris [tris(2,3-dichloropropyl)phosphate] is mutagenic but is currently being used as a replacement for PentaBDE in polyurethane foam products (Rust and Kissinger 2008). Tetrabromobisphenol A (TBBPA) is widely used in electronic equipment and appears to escape less readily into the environment because it is primarily used in a reactive rather than additive mode. However, TBBPA is still found in eggs of predatory birds and in human milk and umbilical cord serum, and it appears to have endocrine-disrupting properties (Legler 2008). Some TBBPA derivatives (ethers) are biologically active, which may lead to health effects.

Unfortunately, the problems with BFRs and CFRs do not stop with their production and use. Uncontrolled burning and dismantling/recycling of electronic and electric waste in developing countries results in contamination and formation of brominated and chlorinated dioxins and furans (Weber and Kuch 2003); these substances are highly toxic, thus causing increased concern both for the health of individuals and for the environment.

The San Antonio Statement is a call for attention to a continuing pattern of unfortunate substitution. Since the 1970s, BFRs and CFRs have commonly served as substitutes for other BFRs and CFRs, even though there have been early warnings and periodic reminders about the problematic properties of these chemicals. To maintain fire safety, safer alternatives to harmful BFRs and CFRs should be developed. In addition, more attention should be paid to the actual need for flame retardants in products. For example, do nursing pillows and baby strollers need flame retardants? Just as we have known for years that significant exposure to lead occurred via house dust, why has it taken us so long to understand that BFRs and CFRs, which are used in consumer products, also can escape their matrix into house, office, car, and airplane dust, and also will end up in people, the environment, and wildlife? Why do we not learn from the past?

The San Antonio Statement represents a reasoned plea from the scientific community to consider the impacts of our use of BFRs and CFRs both for now and for the future.

## Figures and Tables

**Figure:**
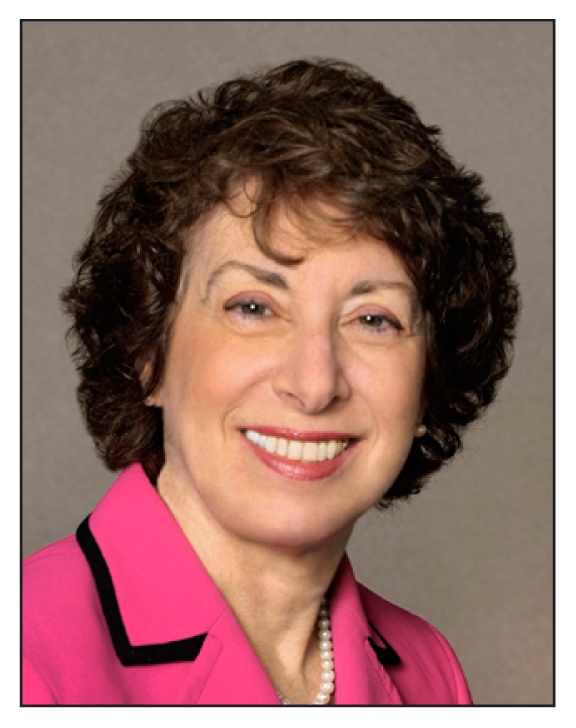
Linda S. Birnbaum

**Figure:**
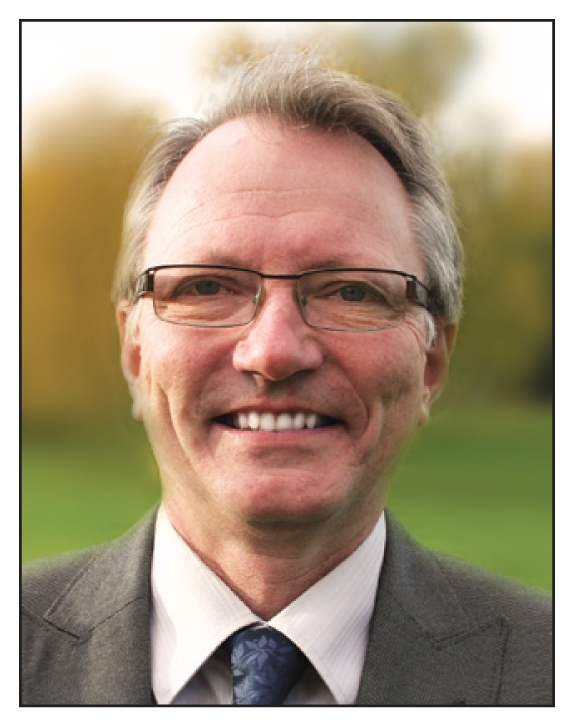
Åke Bergman
